# Thromboelastographic and Gene Polymorphism Bimodality Detection for Dual Antiplatelet Aggregation Therapy in Individuals with Clopidogrel-resistant Symptomatic Intracranial Artery Stenosis

**DOI:** 10.2174/0113862073247573230921102631

**Published:** 2024-02-09

**Authors:** Longlong Liu, Yan Li, Ying Li

**Affiliations:** 1 Department of Emergency, Binzhou People's Hospital, Binzhou, Shandong, 256600, China;; 2 Department of Neurology, Binzhou People's Hospital, Binzhou, Shandong, 256600, China

**Keywords:** Intracranial large artery atherosclerotic stenosis, ischemic stroke, clopidogrel resistance, thromboelastography, clopidogrel gene polymorphism testing, ticagrelor antiplatelet aggregation therapy

## Abstract

**Background:**

Recent research indicates that clopidogrel resistance is connected with a patient's future ischemia risk, hence increasing the likelihood of recurrent ischemic cerebrovascular disease. Thromboelastographic and clopidogrel gene polymorphism testing can be used to see how a person responds to antiplatelet therapy and change the treatment plan accordingly. This may be a good way to make antiplatelet aggregation therapy more effective and safer.

**Objective:**

The objective of this study was to investigate the efficacy of dual antiplatelet aggregation therapy in patients with symptomatic intracranial large artery stenosis being resistant to clopidogrel tablets. The thromboelastographic and gene polymorphism bimodality detection techniques were used to analyze the clopidogrel resistance influencing factors.

**Methods:**

89 patients with symptomatic intracranial large arterial stenosis who were admitted to our hospital from February 2021 to February 2022 were selected, classified as large artery atherosclerotic type by TOAST, and confirmed as having severe intracranial large arterial stenosis (70% to 99%) by magnetic resonance angiography (MRA), computed tomographic angiography (CTA), and digital subtraction angiography (DSA). All patients were treated with dual antiplatelet therapy with aspirin and clopidogrel, and thromboelastography and clopidogrel gene polymorphism were monitored 1 week later.

**Results:**

44 of 89 patients were clopidogrel-resistant. Among 44 patients, 20 were ticagrelor-resistant and 24 were cilostazol-resistant. Clopidogrel had a resistance rate of 49.4%. The recurrence of ischemic cerebrovascular disease in the three groups was statistically significant (*P*<0.05) after 3 months of follow-up treatment, but bleeding (intracranial, gastrointestinal, respiratory, urinary, and mucocutaneous) and dyspnea were not. The clopidogrel-resistant group had a higher number of females, as well as higher levels of hypertension, diabetes, and platelet count than the sensitive group (*P*<0.05), but there was no significant difference in age, smoking, alcohol consumption, previous stroke, glycosylated haemoglobin, creatinine, or low-density cholesterol.

**Conclusion:**

Using thromboelastographic and gene polymorphism bimodality detection, we found switching to ticagrelor antiplatelet aggregation therapy as better than switching to cilostazol in patients with symptomatic intracranial large artery stenosis being resistant to clopidogrel tablets. The results may be biased due to the study being a single-centre study and having a limited sample size.

## 1 INTRODUCTION

Acute ischemic stroke (acute cerebral infarction) is the most common type of stroke, accounting for 69.6% to 70.8% of strokes in China. In China, the 1-year mortality rate of hospitalized patients with acute ischemic stroke is 14.4%~15.4%, and the death/disability rate is 33.4% ~ 33.8% [1, 2]. Intracranial atherosclerotic stenosis (ICAS) is one of the important causes of ischemic stroke, accounting for 30% to 50% of patients with intracranial atherosclerotic stroke in Asian populations. The incidence of intracranial atherosclerosis in Chinese patients with ischemic stroke or transient ischemic attack (TIA) has been reported to be 46.6%, and patients with ICAS have been reported to have more severe symptoms, longer hospital stays, higher stroke recurrence rates, and increased recurrence rates with increasing degrees of stenosis [3, 4].

The 2021 new US guidelines for secondary prevention of stroke and transient ischemic attack (TIA) state that aspirin combined with clopidogrel tablets reduces the risk of recurrent stroke within 90 days in patients with stroke and TIA caused by severe intracranial large artery stenosis (70%-99%) within 30 days [5]. However, even if dual antiplatelet therapy is standardized, 10%-20% of patients still have recurrence after antiplatelet therapy, which is clinically called antiplatelet resistance, and some scholars call it antiplatelet drug unresponsiveness. Among them, clopidogrel resistance is one of the most important reasons leading to substandard antiplatelet therapy in patients [6, 7].

Current studies have shown that the mechanism of clopidogrel resistance is associated with various factors, such as clopidogrel gene polymorphism, drug interactions, patient compliance, abnormal glucose tolerance, and obesity [8, 9]. Current clinical treatment strategies for clopidogrel resistance include increasing the dose of clopidogrel, changing other antiplatelet drugs, and combining other antiplatelet drugs. However, there is no conclusion on which antiplatelet drugs can be replaced for clopidogrel resistance in patients with symptomatic intracranial large artery stenosis, and this issue is discussed in this paper.

The objective of the present study was to examine the effectiveness of dual antiplatelet aggregation therapy in clopidogrel-resistant patients with symptomatic intracranial large artery stenosis using thromboelastographic and gene polymorphism bimodality detection techniques. We have also examined the factors influencing clopidogrel resistance.

## 2 MATERIALS AND METHODS

For clinical data, 89 patients aged 25 to 79 years with symptomatic intracranial large arterial stenosis who were hospitalized in our hospital between February 2021 and February 2022 were chosen. The body weight of the patients in the clopidogrel resistance group (CR) was 67±1.7 kg, and in the clopidogrel sensitive group (CP), it was 65±1.8 kg.

### 2.1 Inclusion Criteria

All patients who have met the diagnostic criteria of the Chinese Guidelines for the Diagnosis and Treatment of Acute Ischemic Stroke (TOAST) classification of large artery atherosclerotic type; brain magnetic resonance angiography (MRA), computer tomography angiography (CTA), or digital subtraction angiography (DSA) have shown symptomatic intracranial large arterial stenosis (more than 70%); a conservative treatment has been selected by the family; an informed consent has been obtained by the patient and the family.

### 2.2 Exclusion Criteria

Those being over the age of 80, or having cardiopulmonary dysfunction, severe liver and kidney dysfunction, or multiple underlying wasting diseases; craniocerebral SWI suggesting microbleeds >10 or cranial DWI suggestive of massive cerebral infarction; history of bleeding and bleeding tendency; high blood pressure variation being difficult to control; poor compliance with oral medications; contraindications to antiplatelet therapy.

### 2.3 Treatment Protocol

According to the Chinese guidelines for secondary prevention of ischemic stroke and transient attack, patients are advised to have a diverse diet with a reasonable intake of energy and nutrients. They are encouraged to consume whole grains, legumes, vegetables, and low-fat dairy products, while reducing the intake of saturated fatty acids and trans fatty acids. All patients admitted to the hospital were given aspirin 100 mg + clopidogrel 75 mg combined with antiplatelet therapy, and atorvastatin was given for intensive treatment and control of cerebrovascular disease risk factors.

All enrolled patients were regularly administered aspirin combined with clopidogrel for 7 consecutive days in the study group. Fasting venous blood was collected in the morning, thromboelastography was completed within 2 hours, and clopidogrel gene polymorphism was assessed. Grouping was based on platelet inhibition rate measured by thromboelastography and clopidogrel gene polymorphism results. Thromboelastography showed the platelet inhibition rate to be <30% due to clopidogrel resistance (CR) and >30% due to clopidogrel sensitivity (CS); clopidogrel gene polymorphism suggested 1/17, 17/17 as ultra-strong metabolic genotype, 1/1 as normal metabolic genotypes, 1/2, 1/3, 2/17 as medium metabolic genotypes, and 2/2, 2/3, and 3/3 as poor metabolic genotype. Patients in the CS group were subjected to thromboelastography, which indicated clopidogrel sensitivity, and clopidogrel gene polymorphism, which indicated ultra-intensive metabolism and a normal metabolic genotype; clopidogrel standard dose plus aspirin 100 mg/day along with antiplatelet aggregation therapy was continued. Thromboelastography suggested clopidogrel resistance, and gene polymorphism suggested patients in the CR group to have moderate and poor metabolic genotypes. The CR group was randomly divided into two groups, *i.e.,* one group was given ticagrelor 90 mg orally, twice daily, combined with aspirin 100 mg orally, once in the evening, along with anti-platelet aggregation therapy, and the other group was given cilostazol 200 mg orally, twice daily, combined with aspirin 100 mg/day and anti-platelet aggregation therapy. Patients were excluded from the study if their thromboelastography and clopidogrel gene polymorphism results did not match, or if their thromboelastography suggested them as being resistant to aspirin. Also, major adverse cerebrovascular events and adverse reactions were determined in the patients. All patients regularly took dual antiplatelet aggregation drugs outside the hospital and were given conventional treatment, such as statins, to control cerebrovascular disease risk factors. Adverse cerebrovascular events and adverse reactions were recorded after 3 months of follow-up. Adverse cerebrovascular events included new ischemic cerebrovascular diseases. Adverse effects included bleeding and dyspnea. Bleeding of different types was observed, including intracranial bleeding, gastrointestinal bleeding, urinary tract bleeding, respiratory bleeding, mucocutaneous bleeding, and so on.

### 2.4 Data Analysis

Baseline data were collected from eligible patients, including demographic information (gender, age, *etc*.), and cerebrovascular disease risk factors (hypertension, diabetes, hyperlipidemia, smoking, drinking, *etc*.). The laboratory tests [low-density lipoprotein (LDL), creatinine (Cr), platelet count, and glycosylated hemoglobin) were conducted in the morning of the next day of admission, and drugs [statins, proton pump inhibitors (PPI), and calcium channel blockers (CCB)] were used after admission.

### 2.5 Statistical Methods

All statistical data were statistically processed using SPSS 26.0 software. Measurement data with a normal distribution have been expressed as x±s, and an independent sample t-test was used to compare groups; enumeration data have been presented in a frequency and percentage table, and the χ2 test was used to compare groups. *P*<0.05 was considered statistically significant.

## 3 RESULTS

Among 89 patients, 44 patients (49.4%) met the criteria for clopidogrel resistance; among them, 24 patients were randomized to receive antiplatelet therapy with cilostazol and 20 patients were randomized to receive ticagrelor.

### 3.1 Clinical Data Between the Clopidogrel Resistance (CR) Group and Clopidogrel Sensitivity (CS) Group

The analysis of basic clinical data for the clopidogrel-resistant group and the clopidogrel-sensitive group is detailed in Table **1**. Among the 89 patients, 44 patients were clopidogrel-resistant, including 20 patients who shifted from clopidogrel to ticagrelor for antiplatelet aggregation, and 24 patients who took cilostazol for antiplatelet aggregation exhibited a clopidogrel resistance rate of 49.4%. The prevalence of female gender, hypertension, diabetes, and platelet count levels were higher in the clopidogrel-resistant group than in the clopidogrel-sensitive group, with a significant difference between the two groups (*P*<0.05). There was no significant difference observed in age, smoking, drinking, previous history of stroke, glycosylated hemoglobin, and low-density cholesterol levels between the two groups (*P* > 0.05).

### 3.2 Adverse Cerebrovascular Events and Adverse Reactions During the 3-Month Follow-Up

Of the 89 patients, 44 were clopidogrel-resistant, including 20 who switched from clopidogrel to ticagrelor for antiplatelet aggregation and 24 who switched to cilostazol for antiplatelet aggregation, with a clopidogrel resistance rate of 49.4%. After 3 months of follow-up treatment, the recurrence of ischemic cerebrovascular disease in the three groups was statistically significant (*P*<0.05); however, there was no statistical significance in bleeding (intracranial bleeding, gastrointestinal tract, respiratory tract, urinary tract, and mucocutaneous bleeding) and dyspnea (Table **2**).

## 4 DISCUSSION

Acute ischemic stroke (acute cerebral infarction) is the most common type of stroke, and intracranial atherosclerotic stenosis (ICAS) is one of the important causes of ischemic stroke. The prevalence of intracranial artery stenosis ranges from 50% to 69%, with an annual stroke risk of 6%, and the prevalence of stenosis ranges from 70% to 99%, with an annual stroke risk of up to 19% [10]. Patients with ICAS have been reported to have more severe symptoms, longer hospital stays, higher stroke recurrence rates, and higher recurrence rates with increasing stenosis. Antiplatelet therapy (APT) significantly reduces mortality or morbidity, reduces recurrence, and reduces the severity of recurrent ischemic stroke. Aspirin and clopidogrel tablets are currently commonly used antiplatelet therapies in clinical practice and are the cornerstone of current ischemic stroke treatment. The new US guidelines for secondary prevention of stroke and transient ischemic attack (TIA) in 2021 stated that within 30 days, antiplatelet therapy with aspirin plus clopidogrel tablets can reduce the risk of recurrent stroke in patients with stroke or TIA caused by severe intracranial large artery stenosis (70%-99%). However, some patients have been reported to not achieve effective antiplatelet therapy after standardized clopidogrel therapy, which is called CR or platelet hyperreactivity (HPR) after antiplatelet therapy [11]. At present, there are different reports on the incidence of clopidogrel resistance in studies. Fu *et al*. [12] used optical turbidimetry to detect platelet function in patients with acute ischemic stroke after taking clopidogrel for 7 days, showing that 48.1% (63/131) of patients still presented platelet HPR after antiplatelet therapy. Kinsella *et al.* [13] employed a platelet function analyzer to detect platelet function in patients with transient ischemic attack after taking clopidogrel treatment and found 92.0% of patients to be clopidogrel-resistant after treatment. Thromboelastography is a rapid and reliable test commonly used in recent years [14]. In this study, thromboelastography was used to detect platelet function in patients with symptomatic intracranial large artery stenosis after 7 days of clopidogrel administration, and the clopidogrel resistance rate was found to be 49.4%.

Clopidogrel is a prodrug, which is an adenosine diphosphate (ADP) receptor P2Y12 antagonist. For many years, the prodrug strategy has been utilized to avoid the occurrence of many unwanted drug properties [15-17]. Clopidogrel is directly esterified to inactive products in about 85% to 90% of cases after intestinal absorption, and only 10% to 15% of cases require conversion to active metabolites by cytochrome P450 isoenzymes in the liver, which inhibit ADP-mediated activation of the glycoprotein (GP) IIb/IIIa complex by irreversibly binding to the ADP receptor P2Y12 on the platelet surface, thereby inhibiting platelet activation and aggregation. The diversity of the antiplatelet response to clopidogrel is influenced by multiple factors, mainly genetic factors, pathophysiological factors, and clinical factors. Many studies have found that variations in genes, such as CYP2C9, CYP3A4 or CYP3A5, CYP2C19, ABCB1, and P2Y12R, in the pharmacokinetic and pharmacodynamic pathways of clopidogrel, are associated with clopidogrel’s antirheumatic effects [18-20].

Among them, CYP2C19 polymorphism is the most common genetic variant associated with clopidogrel resistance. Recent studies have shown carrying one or more CYP2C19 loss-of-function alleles (2* or 3 *) to result in decreased levels of clopidogrel active metabolite *in vivo*, inadequately inhibited platelet aggregation, and increased rates of ischemic events [21, 22]. The CYP2C19 genotype was determined in this study by measuring gene polymorphism in patients in order to determine the type of clopidogrel metabolic rate in patients and platelet reactivity after clopidogrel antiplatelet therapy. Clopidogrel resistance clinical factors include dose and dosage form, medication compliance, drug-related effects, obesity, diabetes, renal insufficiency, and so on [23]. In this study, through the baseline analysis of clinical data and laboratory tests, hypertension, diabetes, and platelet count in the clopidogrel-resistant group were higher than those in the clopidogrel-sensitive group, and the difference between the two groups was statistically significant (*P* < 0.05). There was no significant difference in age, smoking, drinking, and previous history of stroke, glycosylated hemoglobin, creatinine, and low-density cholesterol between the two groups (*P* > 0.05). The study results have been found to be different from those reported in previous studies [24], which may be related to the small sample size in this study, resulting in biased results.

This study has also found that individual differences in responsiveness to antiplatelet therapy are significantly associated with adverse events, such as thrombosis and bleeding. In clinical practice, for patients with clopidogrel resistance, it is necessary to ensure patient compliance, ensure the optimal dose and dosage form of the drug, evaluate drug-drug interactions, and evaluate the patient's infection or inflammatory status. In addition to the above-influencing factors, intensive antiplatelet therapy should be given based on platelet function tests and clopidogrel gene polymorphism tests, including increasing the dose of clopidogrel, changing antiplatelet aggregation drugs, and combining other antiplatelet therapy drugs [8, 24]. A recent study has shown that increasing the clopidogrel dose has a limited effect on improving clopidogrel resistance. González *et al.* [25] showed that in patients after carotid artery stenting, high-dose clopidogrel did not achieve a more potent pharmacodynamic effect than the standard dose at 30 days in patients with platelet hyperresponsiveness to clopidogrel therapy. At present, ticagrelor is a cyclopentyl-triazolopyridine antiplatelet drug that has been studied more frequently in patients with clopidogrel resistance. Compared to clopidogrel, ticagrelor has the characteristics of potent, rapid, and reversible inhibition of platelets, and it can reduce the occurrence of complications, such as gastrointestinal and intracranial hemorrhage. Currently, it is more commonly used in cardiovascular diseases. The PLATO study found that ticagrelor reduced the composite endpoint of all-cause mortality, cardiovascular mortality, myocardial infarction, and stroke compared to clopidogrel, and several studies have shown ticagrelor to have a significant effect on platelet aggregation at a higher dose of clopidogrel in patients with clopidogrel-resistant cardiovascular diseases without increasing the risk of bleeding. According to Malhotra *et al*. [26], ticagrelor may be a useful option for primary and secondary ischemic stroke prevention in patients with vascular risk factors. In 2021, the new US secondary prevention index for stroke and transient ischemic attack (TIA) mentioned that patients who developed a minor stroke or TIA within 24 hours had > 30% stenosis in the ipsilateral large intracranial artery, and 30-day aspirin combined with ticagrelor (90 mg, BID) reduced the risk of stroke recurrence. The results of the PRINCE study showed that aspirin plus ticagrelor significantly inhibited platelets compared to aspirin plus clopidogrel. The SOCRATES trial suggests that more clinical studies are needed to determine whether ticagrelor will replace clopidogrel in the prevention and treatment of ischemic cerebrovascular disease. In this study, among 89 patients, 44 were clopidogrel-resistant, among whom 20 were treated with clopidogrel replaced with ticagrelor for anti-platelet aggregation. There was no difference in the recurrence of ischemic cerebrovascular disease, bleeding, dyspnea, and other adverse events during follow-up after 3 months of treatment between the clopidogrel group and the placebo group. The findings suggest that in patients with clopidogrel resistance, antiplatelet therapy could be replaced with ticagrelor without increasing the risk of bleeding.

Cilostazol is an antiplatelet agent and a phosphodiesterase III (PDE3) inhibitor that improves endothelial function, inhibits platelet aggregation, dilates blood vessels, and mildly inhibits cell growth [27]. In 2021, the new US secondary prevention indicators for stroke and transient ischemic attack (TIA) mentioned that in patients with stroke or TIA caused by 50% to 99% stenosis of the intracranial large arteries, daily cilostazol (200 mg) combined with aspirin or clopidogrel could reduce the risk of stroke recurrence [5, 27].

Na Kagawa *et al*. [28] demonstrated that the addition of cilostazol significantly improved clopidogrel resistance and reduced new ischemic lesions in patients undergoing carotid artery stenting for narrow carotid stenosis without increasing the risk of bleeding.

In this study, however, during the follow-up after 3 months of treatment, cilostazol combined with aspirin antiplatelet aggregation treatment group was found to be statistically significant compared to the clopidogrel group and ticagrelor group in terms of new ischemic cerebrovascular disease, and the results suggested that the cilostazol group had higher new ischemic lesions than the clopidogrel group and the ticagrelor group. No differences in bleeding, dyspnea, *etc*., were observed.

Various studies have shown that clopidogrel resistance is associated with a patient's future ischemic risk, increasing the risk of recurrent ischemic cerebrovascular disease [29-31]. Platelet function testing and clopidogrel gene polymorphism testing can understand individual responsiveness to antiplatelet therapy and adjust the treatment regimen accordingly, which may be an effective means to improve the efficacy and safety of antithrombotic therapy.

## CONCLUSION

In this study, we have investigated the efficacy of dual antiplatelet aggregation therapy in patients with symptomatic intracranial large artery stenosis resistant to clopidogrel tablets using thromboelastography and gene polymorphism bimodality detection. We found switching to ticagrelor antiplatelet aggregation therapy to be superior to switching to cilostazol antiplatelet aggregation therapy for patients with clopidogrel resistance. There are, however, certain limitations involved in this study's representativeness due to the fact that it was conducted at a single centre. Moreover, the sample size was rather small, increasing the likelihood that the results may be biased.

## Figures and Tables

**Table 1:** Comparison of clinical data between the clopidogrel-resistance (CR) group and clopidogrel-sensitivity (CS) group. Normal ranges for laboratory indicators are provided in brackets.

**Basic Information**	**CR Group (n=45)**	**CS Group (n=44)**	***P*-value (t-value)**
**Sex**			
Male	23 (0.51)	32 (0.73)	
Female	22 (0.49)	12 (0.27)	0.036(4.403)
Age (years, x±s)	63.22±9.39	60.66±10.95	0.889(0.020)
Hypertension	39 (0.87)	27 (0.61)	0.006(7.432)
Diabetes	20 (0.44)	10 (0.23)	0.030(4.696)
Hyperlipidemia	12 (0.27)	8 (0.18)	0.338(0.919)
Smoke	17 (0.38)	16 (0.36)	0.89(0.019)
Drinking wine	8 (0.18)	8 (0.18)	0.96(0.002)
Previous stroke history	17 (0.38)	9 (0.20)	0.072(3.228)
**Laboratory Indicators**			
Platelet count (Normal range)	236.51±70.65 (125-350 x 10^9^/L)	198.59±58.83 (125-350 x 10^9^/L)	0.015(2.484) (125-350x 10^9^/L)
Glycosylated hemoglobin (Normal range)	6.3±0.98 (4-6%)	7.0±2.27 (4-6%)	0.093(-1.706) (4-6%)
Creatinine (Normal range)	68±21 (44-133 µmol/L)	74±14 (44-133 µmol/L)	0.545(0.607) (44-133 µmol/L)
Low-density cholesterol (Normal range)	2.53±0.78 (0-3.36 µmol/L)	2.57±0.81 (0-3.36 µmol/L)	0.835(-0.209) (0-3.36 µmol/L)

**Table 2:** Observation of adverse cerebrovascular events and adverse reactions among the three groups.

**Cerebrovascular Events**	**Aspirin+clopidogrel (n=44)**	**Aspirin+Cilostazol (n=24)**	**Aspirin+Tigrenol (n=20)**	***P*-value**
New cerebrovascular disease events (%)	2 (4%)	7 (29.1%)	0	P(Z)=0.023 (7.554)
Hemorrhage	0	0	1 (0.5%)	-
Intracranial hemorrhage	0	0	0	-
Gastrointestinal bleeding	1 (0.02%)	0	0	-
Respiratory tract bleeding	0	0	0	-
Bleeding of the urinary tract	0	0	1 (0.5%)	-
Bleeding of skin and mucous membrane	0	0	0	-
Dyspnea	-	-	-	-

## Data Availability

The datasets used and/or analyzed during the present study are available from the corresponding author, [L.L.].

## LIST OF ABBREVIATIONS

CCB: Calcium Channel Blockers
CP: Clopidogrel Sensitive
CR: Clopidogrel Resistance
Cr: Creatinine
CTA: Computed Tomographic Angiography
DSA: Digital Subtraction Angiography
ICAS: Intracranial Atherosclerotic Stenosis
LDL: Low-Density Lipoprotein
MRA: Magnetic Resonance Angiography
PPI: Proton Pump Inhibitors
TIA: Transient Ischemic Attack
TOAST: Treatment of Acute Ischemic Stroke
